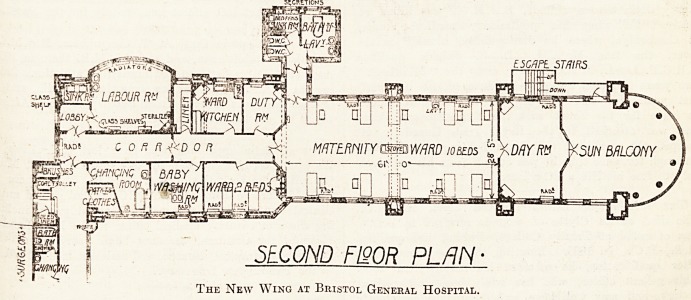# Bristol General Hospital: The Recent Extension

**Published:** 1915-01-09

**Authors:** 


### BRISTOL GENERAL HOSPITAL.

### The Recent Extension.

The new wing designed by Messrs. Oakley and Lawrence, the plan of which we publish below, was described in full detail in our issue of August 1 last (p. 500), on the day of the opening.

The first-floor plan shows a large ward of twenty-two beds, with a spacious semicircular balcony at the south end. This balcony, with that on the floor above, forms a conspicuous and pleasing feature in the exterior. Each floor is supported by gracefully designed stone columns, and the floor of the balcony extends far enough beyond the line of columns to provide an open-air ambulatory.

The sanitary offices are arranged in a well-planned group, projecting from the north side and at the entrance end of the ward.

Two small wards, one for two beds the other for one bed, and a clinical laboratory occupy the south side of the administrative part of the wing, the north side being devoted to duty room, linen store, ward kitchen with larder, patients' clothes store, and three small cupboards labelled "Waste," "Brushes," and "Soiled Linen" respectively. A reference to the article referred to shows that the room, called on the plan "Duty Room," is really a sister's room; without this explanation it is difficult to understand why there should be both ward kitchen and duty room, which are only two names for the same thing.

The second-floor plan is the maternity department, and contains a ward for ten beds, with a day room and balcony, and sanitary offices exactly like those below. There are in addition a ward for two beds, baby-washing room, nurses' changing room, and store for patients' clothes on the south side; with sisters' rooms, ward kitchen, linen store, and labour room on the north side.

A changing room and bathroom are also provided for the surgeons.

The New Wing at Bristol General Hospital.

## Figures and Tables

**Figure f1:**
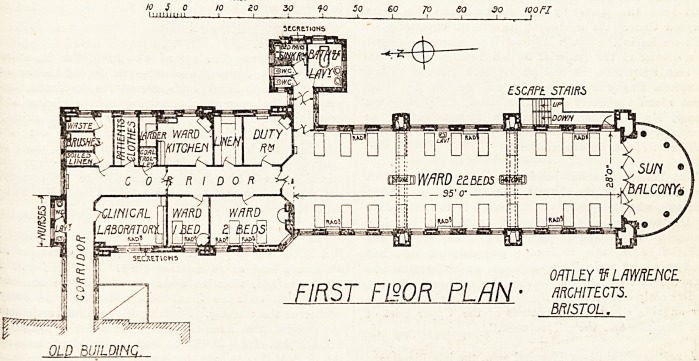


**Figure f2:**